# Detection methods predict differences in biology and survival in breast cancer patients

**DOI:** 10.1186/1471-2407-12-604

**Published:** 2012-12-17

**Authors:** Maximino Redondo, Rafael Funez, Francisco Medina-Cano, Isabel Rodrigo, Mercedes Acebal, Teresa Tellez, M Jose Roldan, M Luisa Hortas, Ana Bellinvia, Teresa Pereda, Laia Domingo, María Morales-Suarez Varela, Maria Sala, Antonio Rueda

**Affiliations:** 1 Research Unit, Hospital Costa del Sol, University of Málaga, Red de Investigación en Servicios de Salud en Enfermedades Crónicas (REDISSEC), Carretera de Cádiz Km 187, 29600, Marbella, Málaga, Spain; 2 Department of Pathology, Hospital Costa del Sol, 29600, Marbella, Málaga, Spain; 3 Department of Surgery, Hospital Costa del Sol, 29600, Marbella, Málaga, Spain; 4 Department of Radiology, Hospital Clinico Universitario Virgen de la Victoria, Campus Universitario Teatinos, 29010, Málaga, Spain; 5 Department of Radiology, Hospital Costa del Sol, 29600, Marbella, Málaga, Spain; 6 Epidemiology and Evaluation Department. IMIM Hospital del Mar, Red de Investigación en Servicios de Salud en Enfermedades Crónicas (REDISSEC). Parc de Salut Mar, Universitat Autònoma de Barcelona, Catalonia, Spain; 7 Unit of Public Health and Environmental Care, Department of Preventive Medicine, CIBER ESP, University of Valencia, Valencia, Spain; 8 Department of Medical Oncology, Hospital Costa del Sol, Red de Investigación en Servicios de Salud en Enfermedades Crónicas (REDISSEC), 29600, Marbella, Málaga, Spain

**Keywords:** Breast cancer, Detection methods, Proliferation, Apoptosis, Survival

## Abstract

**Background:**

The aim of this study was to measure the biological characteristics involved in tumorigenesis and the progression of breast cancer in symptomatic and screen-detected carcinomas to identify possible differences.

**Methods:**

For this purpose, we evaluated clinical-pathological parameters and proliferative and apoptotic activities in a series of 130 symptomatic and 161 screen-detected tumors.

**Results:**

After adjustment for the smaller size of the screen-detected carcinomas compared with symptomatic cancers, those detected in the screening program presented longer disease-free survival (RR = 0.43, CI = 0.19-0.96) and had high estrogen and progesterone receptor concentrations more often than did symptomatic cancers (OR = 3.38, CI = 1.72-6.63 and OR = 3.44, CI = 1.94-6.10, respectively). Furthermore, the expression of bcl-2, a marker of good prognosis in breast cancer, was higher and HER2/neu expression was lower in screen-detected cancers than in symptomatic cancers (OR = 1.77, CI = 1.01-3.23 and OR = 0.64, CI = 0.40-0.98, respectively). However, when comparing prevalent *vs* incident screen-detected carcinomas, prevalent tumors were larger (OR = 2.84, CI = 1.05-7.69), were less likely to be HER2/neu positive (OR = 0.22, CI = 0.08-0.61) and presented lower Ki67 expression (OR = 0.36, CI = 0.17-0.77). In addition, incident tumors presented a shorter survival time than did prevalent ones (RR = 4.88, CI = 1.12-21.19).

**Conclusions:**

Incident carcinomas include a variety of screen-detected carcinomas that exhibit differences in biology and prognosis relative to prevalent carcinomas. The detection method is important and should be taken into account when making therapy decisions.

## Background

The widespread introduction of mammographic screening for breast cancer has led to a 20% reduction in breast cancer mortality [1]. Tumors detected by mammographic screening are generally considered to have good prognoses because of several biases, such as selection bias, lead-time bias, length bias, and, possibly, overdiagnosis (some tumors might never have surfaced) [2]. In support of these observations autopsy studies have revealed occult breast cancer in 1.3% and in situ carcinoma in 8.9% –18% of women [3,4]. However, it is impossible to determine the natural history of these tumors. Screening will inevitably detect a greater proportion of slower-growing, better-prognosis cancers than those observed in the symptomatic population. The remainder of the survival advantage is likely to be due to additional biological differences between screen-detected and symptomatic cancers, including rates of hormone receptor positivity and proliferation and other biological factors [5].

Therefore, screening enables the detection of breast cancers at an earlier stage of disease. It is now well documented that screen-detected cancers are generally smaller, of lower grade and less likely to have axillary lymph node involvement [6].

A number of prognostic factors for symptomatic breast cancer have been identified. The important pathological features of prognostic significance are tumor size and the presence of lymph node metastases. In symptomatic breast cancer, the presence of lymph node metastases is generally considered to be the most important prognostic factor. In screen-detected cancers, however, the incidence of tumors with lymph node involvement is low because of earlier diagnosis and the smaller size of the tumor and is, therefore, of limited prognostic value. Fixing the size of the tumor (by comparing same-size tumors) reduces the lead-time bias. More important is the possible impact of length bias. It is theoretically possible to eliminate length bias by adjusting for the aggressiveness of tumors on the basis of a full biological description [5,7,8].

At present, cancer detection based on mammography screening is not considered to be of significant importance when assessing the risk of breast cancer recurrence or in decision making concerning the need for adjuvant therapies in the diagnosis of early breast cancer. If cancerous tumors detected by mammography screening were associated with better outcomes than tumors of similar size detected by methods other than screening, women with a lower risk of recurrence might be subjected to adjuvant therapies. Recent studies have shown that screen detection remains an independent prognostic factor after adjusting for disease stage [5,7-9].

This paper, therefore, examines whether a cancer detected by mammographic screening confers additional prognostic benefit to the patient over and above that expected by the improved stage shift. In addition, the pathologic features of breast cancer diagnosed in a first screening round (prevalent) were compared with those of incident cases on the basis that prevalent cancers have a potentially longer period over which to develop prior to detection than incident cancers, for which this period is theoretically limited by the screening interval.

Several studies have reported that tumors detected between mammography screening rounds (interval cancers) are similar to those found outside screening programs [10,11]. However, it has not been shown that a cancer detected at a round subsequent to one or more screens (incident tumors) has a different biology and outcome from tumors detected in the first round (prevalent tumors) or outside the screening program.

## Material and methods

### Patients and samples

A nested case–control study was performed among woman who were histologically diagnosed with breast cancer and who had undergone surgical resection between 1996 and 2007. Thus, the study population consisted of 291 patients referred to the symptomatic (n = 130) and screening (n = 161) services of Costa del Sol Hospital. The symptomatic clinic was attended by patients who were referred with breast abnormalities, typically palpable lesions, by their primary care physicians. There were no differences in mean patient age between screen-detected and symptomatic carcinomas (56.33 ± 1.14 vs. 56.73 ± 1.18) (Table 1).

**Table 1:** Clinical-pathological prognostic features of disease

	**Screen-detected (n = 161) vs. Symptomatic (n = 130)**	**P value**	**OR adjusted by tumor size**
**Mean age**	56.33 ± 1.14 vs. 56.73 ± 1.18	N.S.	1.03 (0.99-1.05)
**Mean size (cm)**	1.62 + 0.14 vs. 2.68 + 0.15	p < 0.001	--
**Lymph node positive**	22.3% vs. 48.9%	p < 0.001	0.54 (0.31-0.95)
**Poorly differentiated tumors**	33.1% vs. 39.7%	N.S.	0.75 (0.47-1.20)
**ER positive**	87.8% vs. 57.4%	p < 0.001	4.76 (2.50-9.09)
**PR positive**	73.1% vs. 36.4%	p < 0.001	4.16 (2.43-7.14)
**In situ carcinomas**	11.4% vs.1.5%	p < 0.001	6.25 (1.35-33.3)
**Apoptosis**	48.8% vs. 38.5%	N.S.	1.53 (0.84-2.77)
**Bcl-2**	78.4% vs. 62.7%	p < 0.01	1.92 (1.08-3.44)
**c-erb-B2**	14.6% vs. 26.3%	p < 0.05	0.57 (0.15-0.97)
**Ki67**	46.7% vs. 52.5%	N.S.	0.97 (0.57-1.63)
**Delay Diagnosis- treatment (>30 days)**	47.5% vs. 57%	N.S.	0.75 (0.46-1.25)

ER: estrogen receptor, PR: progesterone receptor.

In general, following an initial screen, women are invited to attend rescreening at intervals of two years, while annual screens are offered to individuals with a clinical indicator of increased breast cancer risk detected at screening or who had a first-degree relative with a history of breast cancer. This study was approved by the Costa del Sol Hospital ethics committee, and informed consent was obtained to supply tumor material for pathologic evaluation and immunohistochemical analyses.

Cancers diagnosed at the initial screening episode were designated as prevalent. Cancers diagnosed at a round subsequent to one or more screens in which cancer was not detected were classed as incident. All mammograms were reviewed to confirm incident tumors. In our series, there were 76 prevalent tumors, 65 incident tumors, and we also considered a third group, false negative mammograms, which included those tumors that were present in the first round but were not detected (n = 20).

Interval cancers, defined as cancers detected within 24 months after a negative mammogram but before the following invited screening, were excluded from this study. Furthermore, patients who received pre-operative adjuvant therapy were excluded from the study. In situ carcinomas were also excluded from the survival analysis.

In the survival studies, our primary endpoint was time to recurrence or breast cancer-specific death as measured from the time of diagnosis. Survival times of patients still alive or who died of other causes were censored as of the date of the last follow-up. Follow-up was conducted by the Hospital Tumor Registry and was achieved for 93.7% of patients with a median follow-up period of 71 months.

Finally, we considered treatment delay, which was defined as a delay of longer than one month between diagnosis and the first treatment.

All specimens were fixed in 10% neutral buffered formalin and embedded in paraffin. The following clinical and histopathological data were collected from all cases: patient age at diagnosis, differentiation degree, hormonal receptor status, tumor size, presence of regional (lymph node) metastases, tumor stage and patient survival. Pathological staging was performed according to the postsurgical International Union Against Cancer Tumor-Node-Metastasis classification. The histological typing and grading of the tumors was performed according to the World Health Organization classification. Two hundred and thirty cases were ductal adenocarcinomas and 61 tumors belonged to other histological subtypes.

### HercepTest IHC assay

In this study, HER2/neu protein expression was evaluated using the HercepTest for Immunoenzymatic Staining at PPL according to the protocol described in the manufacturer's guide accompanying the kit. Tissue sections were deparaffinized in two 5-minute changes of xylene and were rehydrated using a gradient of alcohols culminating in distilled water. Subsequently, the slides were immersed in Dako Epitope Retrieval Solution (Dako, Copenhagen, Denmark, 0.01 mol/L citrate buffer; pH = 6) that had been preheated to 95°C; then, this solution was heated in a water bath at 95°C for a total of 40 minutes, followed by a 20-minute cool-down period at room temperature. The slides were incubated with the primary rabbit polyclonal antibody to the HER2/neu oncoprotein (as supplied prediluted in the HercepTest kit) on a Dako Autostainer for 30 minutes at room temperature. The antibody was localized by incubating the slides with the Dako Visualization Reagent (dextran polymer conjugated with horseradish peroxidase and goat anti-rabbit immunoglobulins) for 30 minutes using the Dako Autostainer. Diaminobenzidine (DAB) was used as the chromogen, and the sections were counterstained with hematoxylin. Positive controls were included in each staining run and consisted of freshly cut breast cancer cases known to express HER-2/neu and a control slide consisting of three pelleted, formalin-fixed, paraffin-embedded human breast cell lines with staining intensity scores of 0, 1+, and 3+ (supplied in the HercepTest kit). Negative controls consisted of substituting normal rabbit serum (Dako Negative Control Reagent) for the HER-2/neu primary antibody. Only membrane staining intensity and pattern were evaluated using the 0 to 3+ scale, as illustrated in the HercepTest kit scoring guidelines. As defined in the HercepTest kit guide, scores of 0 or 1+ were considered negative for HER2/neu overexpression, 2+ was considered weak positive, and 3+ was considered strong positive. To qualify for 2+ and 3+ scoring (i.e., positive), complete membrane staining of more than 10% of tumoral cells had to be observed.

We also used a modification of this scoring system that took into consideration the level of staining of nonneoplastic epithelium present on the same slide as the cancer. In this system, nonneoplastic epithelium was also graded on a 0 to 3+ scale using the same criteria used for the assessment of tumoral cell staining. Cases were considered HER2/neu positive only when the difference between the tumoral cell staining score and the nonneoplastic epithelial cell staining score was 2.

### Immunohistochemistry

We studied tumor proliferation and the expression of hormone receptors and proteins related to the apoptotic process by detecting the expression of Ki67, HER2/neu, estrogen receptors, progesterone receptors and bcl-2 (Dako, Copenhagen, Denmark).

One representative block from each patient was sectioned at 5 μm and stained with the primary antibody. A standard, three-step technique using an avidin-biotin-complex/horseradish peroxidase (HRP) kit (Dako) was used as described previously [12]. For the negative control, the primary antibodies were replaced with phosphate-buffered saline (PBS). Tumors and tissues with known staining patterns were used as positive immunostaining controls. Mononuclear infiltrates were used as positive internal controls for bcl-2.

Protein expression was analyzed in 20 different fields of the tumors, and the reported values represent the means of the areas measured. Expression was scored as follows: negative if no staining was observed or if immunoreactivity was observed in less than 10% of the tumor cells and positive if more than 10% of the tumor cells showed staining with an intensity >1 (maximum value = 3). Samples were analyzed and scored blindly.

Scoring was performed by two independent observers, and discrepant results were discussed over a double-headed microscope.

### In situ localization of apoptotic cells

To detect apoptotic cells, in situ labeling of the 3'-ends of the DNA fragments generated by apoptosis-associated endonucleases was performed using a commercial apoptosis detection kit (Roche Diagnostic, Germany). Briefly, deparaffinized sections were incubated with 20 μg/ml of proteinase K (Sigma Chemical Co. St. Louis, MO) for 15 min. Following rinsing in PBS, the slides were covered with terminal deoxynucleotidyl transferase plus a nucleotide mixture at a 1:35 dilution for 60 min at 37°C. Then, the slides were covered with an anti-fluorescein antibody conjugated with alkaline phosphatase. After substrate reaction, the stained cells were analyzed under a light microscope. Pretreatment of sections with DNase served as a positive control for the enzymatic procedures; omission of the enzyme served as a negative control.

Established morphological features used to identify apoptosis on H&E were also required in TUNEL-stained slides. Cells were defined as apoptotic if the entire nuclear area of the cell was positively labeled. Apoptotic bodies were defined as small, positively labeled globular bodies in the cytoplasm of the tumor cells that could be found either singularly or in groups.

One thousand cells were counted for each specimen. The number of positively stained cells was then divided by 1000 to estimate the percentage of apoptotic cells in each specimen. We used the mean level of apoptosis in our series (1%) (range 0.01-10.8%) as a cut-off.

### Statistical analysis

Differences between the detection groups with regard to patient characteristics, including clinical, biological and histopathological variables, were analyzed via cross-tabulation (Fisher’s exact test) for categorical variables and analysis of variance for continuous variables (natural log transformed when necessary). Survival curves were generated using the Kaplan-Meier method, and statistical testing was performed using the log-rank test. Multivariate analyses and HR calculations with 95% CIs were performed using the Cox proportional hazards model. All computations were executed using SPSS software (Chicago, IL). All P values are two-sided.

## Results

### Symptomatic versus screen-detected carcinomas

Screen-detected patients had persistently smaller tumors (1.62 ± 0.14 *vs*. 2.68 ± 0.15, p < 0.001), a lower rate of axillary node metastases (22.3% *vs*. 48.9%, p < 0.001) and a higher percentage of in situ carcinomas (11.4% *vs*. 1.5%, p < 0.001). We also found biological differences between the two groups. Screen-detected tumors were more frequently estrogen receptor and progesterone receptor positive (87.8% *vs*. 57.4%, p < 0.001 and 73.1% *vs*. 36.4%, p < 0.001, respectively), presented a higher expression of bcl-2 protein (78.4% *vs*. 62.7%, p < 0.01) and were less frequently HER2/neu positive (14.6% *vs*. 26.3%, p < 0.05) (Table 1). These relationships were maintained when in situ carcinomas were excluded from the analysis (data not shown).

After adjustment for the smaller size of the screen-detected primary tumors compared with control cancers, the differences between the two groups were maintained and related to axillary nodal metastases (OR = 0.25; CI = 0.13-0.47), percentage of in situ carcinomas (OR = 6.19, CI = 1.35-28.37) and estrogen and progesterone receptor expression (OR = 3.38, CI = 1.72-6.63; OR = 3.44, CI = 1.94-6.10, respectively). Furthermore, the expression of bcl-2 was higher (OR = 1.77, CI = 1.01-3.23) and that of HER2/neu was lower in screen-detected cancers compared with symptomatic ones (Table 1).

When prevalent and incident tumors were separated and compared with symptomatic tumors (Tables 2 and 3), similar results were obtained. However, incident screen-detected patients were older (OR = 1.04 CI = 1.008-1.081), and their tumors exhibited apoptotic activity more frequently (OR = 5.06; CI = 1.71-14.94). Additionally, the treatment delay was short in the case of incident screen-detected carcinomas (OR = 0.47, CI = 0.23-0.95) (Table 2).

**Table 2:** Clinical-pathological prognostic features of disease

	**Incident (n = 65) vs. Symptomatic (n = 130)**	**P value**	**OR adjusted by tumor size**
**Mean age**	59.7 ± 0.68 vs. 56.3 ± 1.14	p < 0.05	1.04 (1.01-1.08)
**Mean size (cm)**	1.27 ± 0.17 vs. 2.68 ± 0.15	p < 0.001	--
**Lymph node positive**	21.5% vs. 48.9%	p < 0.001	0.78 (0.34-1.76)
**Poorly differentiated tumors**	32.8 vs. 39.7%	N.S.	0.56 (0.27-1.17)
**ER positive**	89.1% vs. 57.4%	p < 0.001	4.23 (1.61-11.12)
**PR positive**	78.2 vs. 36.4%	p < 0.001	4.60 (2.11-10.03)
**In situ carcinomas**	11% vs. 1.5%	p < 0.001	2.44 (0.42-13.87)
**Apoptosis**	66.7% vs. 38.5%	p < 0.05	5.06 (1.71-14.94)
**Bcl-2**	81.3% vs. 62.7%	p < 0.05	1.39 (0.89-2.19)
**c-erb-B2**	26.3% vs. 23%	N.S.	0.86 (0.21-3.53)
**Ki67**	63% vs. 52.5%	N.S.	1.96 (0.93-4.09)
**Delay Diagnosis- treatment (>30 days)**	35.4% vs. 57%	p < 0.05	0.47 (0.23-0.95)

**Table 3:** Clinical-pathological prognostic features of disease

	**Prevalent (n = 76) vs. Symptomatic (n = 130)**	**P value**	**OR adjusted by tumor size**
**Mean age**	56.8 ± 0.65 vs. 56.3 ± 1.14	N.S.	1.01 (0.98-1.03)
**Mean size (cm)**	1.64 ± 0.15 vs. 2.68 ± 0.15	p < 0.001	--
**Lymph node positive**	25% vs. 48.5%	p < 0.001	0.54 (0.27-1.08)
**Poorly differentiated tumors**	32.1% vs. 39.7%	N.S.	0.55 (0.29-1.04)
**ER positive**	87.5% vs. 57.4%	p < 0.001	4.56 (1.98-10.52)
**PR positive**	70.3% vs. 36.4%	p < 0.001	3.65 (1.88-7.08)
**In situ carcinomas**	13% vs. 1.5%	p < 0.001	8.30 (1.70-40.31)
**Apoptosis**	42.5% vs. 38.5%	N.S.	1.45 (0.67-3.16)
**Bcl-2**	75.4% vs. 62.7%	N.S.	1.52 (0.75-3.06)
**c-erb-B2**	7.4% vs. 26.3%	p < 0.01	0.59 (0.31-0.90)
**Ki67**	38.5 vs. 52.5%	N.S.	0.64 (0.34-1.22)
**Delay Diagnosis-treatment (>30 days)**	64.5% vs. 55%	N.S.	1.51 (0.81-2.81)

### Prevalent versus incident screen-detected carcinomas

Comparison of prevalent versus incident screen-detected carcinomas (Table 4) showed that in prevalent carcinomas, the tumors were larger (1.64 ± 0.15 *vs.* 1.27 ± 0.17; p = 0.04) and the patients were younger (56.88 ± 0.65 *vs.* 59.71 ± 0.68, p < 0.01); the presence of lymph node metastases did not differ between prevalent and incident-detected cancers (Table 4). However, prevalent tumors were less likely to be HER2/neu and Ki67 positive (O.R = 0.22; CI = 0.087-0.61 and OR = 0.36, CI = 0.17-0.77, respectively) and presented a longer delay prior to receiving treatment after diagnosis (OR = 3.31; CI = 1.65-6.62).

**Table 4:** Clinical-pathological prognostic features of disease

	**Prevalent (n = 76) vs. Incident screened (n = 65)**	**P value**	**OR**
**Mean age**	56.88 ± 0.65 vs. 59.71 ± 0.68	P < 0.01	0.91 (0.86-0.98)
**Mean size (cm)**	1.64 ± 0.15 vs. 1.27 ± 0.17	p < 0.05	1.44 (1.01-2.08)
**Lymph node positive**	25% vs. 21.5%	N.S.	1.21 (0.55-2.66)
**Poorly-differentiated tumors**	32.1% vs. 32.8%	N.S.	0.96 (0.49-1.90)
**ER positive**	87.5% vs. 89.1%	N.S.	0.85 (0.27-2.64)
**PR positive**	70.3% vs. 78.2%	N.S.	0.66 (0.28-1.52)
**in situ carcinomas**	13% vs. 11%	N.S.	1.25 (0.44-3.51)
**Apoptosis**	42.5% vs. 66.7%	N.S.	0.37 (0.12-1.11)
**Bcl-2**	75.4% vs. 81.3%	N.S.	0.70 (0.28-1.77)
**Ki67**	38.5% vs. 63%	p < 0.01	0.36 (0.17-0.77)
**c-erb-B2**	7.4% vs. 23%	p < 0.01	0.22 (0.08-0.61)
**Delay Diagnosis- treatment (>30 days)**	64.5 vs. 35.4	p < 0.01	3.31 (1.65-6.62)

In our series, we detected 20 cases with a previous false negative mammogram. In spite of the small number of such cases, we found statistically significant differences in the percentage of cells that expressed Ki67 antigens. Only 33% were positive in the group of false negative mammograms versus 63% for the true incident screen-detected carcinomas (OR = 0.29; CI = 0.08-0.98) (Table 5).

**Table 5:** Clinical-pathological prognostic features of disease

	**False Negative mammograms (n = 20) vs. Incident (n = 65)**	**P value**	**OR**
**Mean age**	60.8 + 1.1 vs. 59.7 + 0.6	N.S.	1.04 (0.93-1.15)
**Mean size (cm)**	1.17 + 0.16 vs. 1.27 + 0.09	N.S.	0.79 (0.34-1.86)
**Lymph node positive**	6.3% vs. 21.5%	N.S.	0.24 (0.02-2.00)
**Poorly differentiated tumors**	50% vs. 40%	N.S.	1.50 (0.50-4.49)
**ER positive**	95% vs. 89%	N.S.	1.59 (0.17-14.4)
**PR positive**	85.7% vs. 78.2%	N.S.	1.67 (0.32-8.52)
**In situ carcinomas**	6.3% vs. 11%	N.S.	0.55 (0.06-4.84)
**Apoptosis**	42.9% vs. 66.7%	N.S.	0.37 (0.06-2.15)
**Bcl-2**	84.6% vs. 81.3%	N.S.	1.26 (0.23-6.75)
**c-erb-B2**	15% vs. 23.1%	N.S.	0.41 (0.04-3.79)
**Ki67**	33.3% vs. 63%	p < 0.05	0.29 (0.08-0.98)
**Delay Diag- treat (>30 days)**	37.9% vs. 35.4%	N.S.	1.09 (0.33-3.59)

ER: estrogen receptor, PR: progesterone receptor.

### Survival by method of detection

Screen-detected carcinomas had the longest survival period. This result was expected because the comparison of survival time would be affected by lead time and other biases. To minimize lead-time bias in the following analyses, we compared survival distributions by method of detection for patients whose breast cancers were the same size. After adjusting for tumor size, we found that screen-detected carcinomas presented a decreased percentage of recurrences and better disease-free survival. Thus, for tumors ≤ 2 cm, the percentage of recurrence was 30% for symptomatic tumors, while it was only 6% for screen-detected tumors (p < 0.05). When we selected tumors > 2 cm, the percentages were 39% and 14%, respectively (p < 0.05). Disease-free survival adjusted by tumor size relative risk (RR) was 0.33 (CI = 95%: 0.15-0.70).

When we introduced not only tumor stage but also biological characteristics into the multivariate analysis, the method of detection maintained its prognostic value (RR = 0.42; CI = 0.19-0.93).

**Figure 1:**
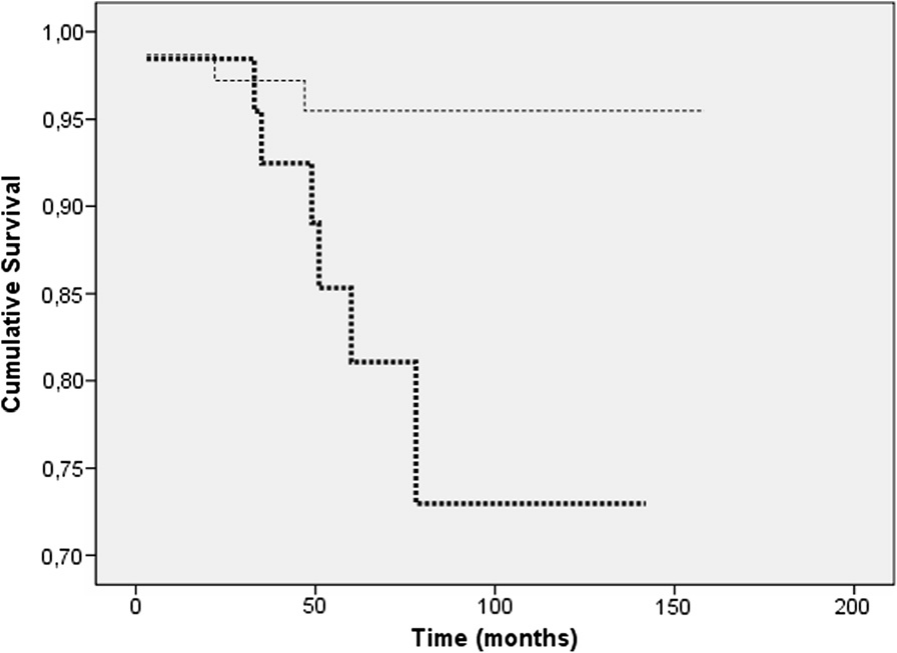
**Disease-specific survival distribution in screen-detected carcinomas**. The cumulative survival of patients in the incident group (thick line) is significantly shorter than that of patients in the prevalent group (thin line).

Comparison of prevalent vs. incident carcinomas showed that survival was significantly shorter for incident cases (RR = 4.88, CI = 1.12-21, 19) (Figure 1). No differences in survival were detected between incident cases and symptomatic ones (RR = 0.57, CI = 0.46-3.96). However, when we compared prevalent vs. symptomatic carcinomas, survival was found to be significantly longer for prevalent cases (OR = 0.34, CI 0.13-0.88). Therefore, incident carcinomas constitute a type of screen-detected carcinoma that exhibits a worse prognosis than prevalent carcinomas.

No event was detected in the 20 cases of false negative mammograms.

## Discussion

We found the method of detection to be an important prognostic factor for breast cancer survival, even after adjusting for tumor characteristics.

Because lead time manifests itself as an earlier stage of disease, fixing the stage of disease reduces the magnitude of lead-time bias. Such an adjustment, however, has little or no effect on length bias. Cancers found via screening include a higher proportion of slowly growing tumors, some of which might never be found by other means; this observation represents an extreme form of length bias known as overdiagnosis bias. Some studies indicate that the disease prognosis may be predestined at the time of diagnosis, independent of the tumoral characteristics at diagnosis [13,14]. The other biological characteristics are potentially critical factors that determine the aggressiveness of a tumor and, thus, could be used to further quantify the length bias. The established profile of aggressive breast tumors includes metastasis to regional lymph nodes, loss of ERs and PRs, high proliferative rate and overexpression of c-erbB-2 oncogene [15,16]. However, apoptosis has a strong association with proliferation, and in previous studies, apoptosis in primary breast carcinomas was independently associated with shorter survival [17,18]. However, bcl-2 expression was statistically associated with a better clinical outcome and with a number of favorable prognostic features [19-21]. Our results indicate that screen-detected breast carcinomas are significantly associated with several features indicative of low malignant potential, as has been described in other studies [9,22,23].

The generally favorable outcomes of women with cancerous tumors detected by mammography screening compared with women whose tumors were found by other means might be explained not only by the smaller tumor size detected by screening but by the more favorable biological features of these tumors. In our series, cancerous tumors detected by screening were more often HER2/neu negative. In addition, bcl-2 and estrogen and progesterone receptors were found to be positive at a significantly higher rate in screen-detected tumors than in symptomatic tumors. Similar findings were reported by Crosier et al. [24] and Dawson et al. [9]. However, these features do not fully explain the generally better outcomes of women with cancerous tumors detected by mammography screening because the mode of detection was an independent prognostic variable in the multivariate analyses.

In the present study, breast carcinoma recurrence rates were significantly lower among screened patients compared with unscreened patients after adjusting for tumor size. Two previous studies [25,26] have also reported significant differences in 5-year recurrence rates between screened and unscreened women. In addition, our conclusions support those of other studies [5,9] in showing that the method of detection is an independent prognostic factor. As in the study by Joensuu et al. [5], we adjusted the outcome for tumor size, the number of axillary lymph nodes involved, tumor grade and hormone receptor content, as well as for prognostic factors, such as Her-2 status and Ki67. Even after adjusting for all of these factors that might be expected to reflect the aggressiveness of tumor growth (and, hence, length bias due to screening), we found that diagnosis by a method other than mammographic screening was a statistically significant independent predictor of short disease-free survival. These results would appear to confirm those of previous studies that have suggested that screen detection is an independent prognostic factor for both disease-specific survival [7-9] and distant recurrence [5]. Our results also show that in tumors < 2 cm, the disease-specific survival of symptomatic cancer is shorter than that in the screen-detected group. In fact, Joensuu et al. [5] observed that in women aged 50–69 years with node-negative tumors, the 10-year distant disease-free survival rate was better in the screen-detected cohort than in the symptomatically presenting group (93% vs. 87% for tumors ≤1 cm). This survival benefit is most likely due to differences in tumor biology between screen-detected and symptomatic cancers.

Interestingly, this study clearly shows that incident cancers are biologically different from prevalent ones. No previous studies have measured the expression of biological markers of prognosis in incident cancers. Incident cancers appeared to have worse prognosis than that of prevalent cancers based on the expression of biological marker. Additional tumor characteristics commonly associated with aggressive clinical behavior in breast cancer, such as positivity for Ki67 and HER2/neu, were associated with incident-detected cancers, which supports the hypothesis that incident cancers are biologically more aggressive than their prevalent screen-detected counterparts. Clearly, however, the size of breast cancers increases with time, as prevalent cancers were larger than incident cancers. The poorer outcomes for incident cancers may be associated with their biologic differences and more rapid tumor growth. In fact, these incident carcinomas were found after a “normal” mammogram, which suggests a faster growth rate for these tumors. It is known that indices of rapid growth are associated with breast cancer aggressiveness and poorer prognosis [27,28].

It is possible that cancers diagnosed at the first screening round are subject to overdiagnosis and length bias; thus, first-round screening may have a lesser effect on breast cancer mortality. We also considered incident cancers separately from cancers found in the symptomatic group. Nevertheless, we found similar survival distributions for incident cancers and cancers detected in the symptomatic group.

In a previous work [29], a short, in-hospital, diagnostic delay for breast carcinomas was associated with advanced disease state and poor survival. In this series of patients, we report a short treatment delay for incident tumors. This delay most likely indicates that doctors give priority to patients with a previous negative mammogram.

## Conclusions

We conclude that prevalent screen-detected breast cancer is associated with a favorable prognostic profile. Physicians should know that patients whose breast cancers are detected in the first round of screening have a higher probability of a better prognosis. Current treatment paradigms do not consider the method of tumor detection to be important when selecting systemic adjuvant therapies. We feel that the mode of breast cancer detection should be taken into account when determining individual patient management strategies. Studies with larger series of patients are needed to corroborate our findings and to identify new biological characteristics associated with the prognosis of screen-detected carcinomas.

## Abbreviations

ERs: Estrogen receptors; PRs: Progesterone receptors; TUNEL: Tdt-mediated dUTP Nick End Labeling; HR: Hazard Ratio; OR: Odds Ratio.

## Competing interests

The authors declare that they have no competing financial interests.

## Authors’ contributions

**MR**: Conceived of the study, performed the statistical analysis, and drafted the manuscript. **RF and IR**: Evaluated immunohistochemical and apoptosis detection techniques. **FMC**: Acquisition and interpretation of data. **MA and AB**: Reviewed and classified the mammograms. **TT and MJR**: Performed the TUNEL technique. **MLH and LD**: Participated in immunohistochemical investigations. **MMSV**, **MS and AR**: Participated in the design of the study, interpretation of the results and drafting of the manuscript. All authors read and approved the final manuscript.

## Pre-publication history

The pre-publication history for this paper can be accessed here:

http://www.biomedcentral.com/1471-2407/12/604/prepub
